# The Frequency and Severity of Complications in Surgical Treatment of Osteochondral Lesions of the Talus: A Systematic Review and Meta-Analysis of 6,962 Lesions

**DOI:** 10.1177/19476035231154746

**Published:** 2023-03-09

**Authors:** Julian J. Hollander, Jari Dahmen, Kaj S. Emanuel, Sjoerd A.S. Stufkens, John G. Kennedy, Gino M.M.J. Kerkhoffs

**Affiliations:** 1 Department of Orthopaedic Surgery and Sports Medicine, Amsterdam Movement Sciences, Amsterdam UMC, Location AMC, University of Amsterdam, Amsterdam, The Netherlands; 2 Academic Center for Evidence Based Sports Medicine, Amsterdam UMC, Amsterdam, The Netherlands; 3 Amsterdam Collaboration for Health and Safety in Sports, International Olympic Committee Research Center, Amsterdam UMC, Amsterdam, The Netherlands; 4 Department of Orthopedic Surgery, Maastricht University Medical Center+, Maastricht, The Netherlands; 5 Department of Orthopedic Surgery, NYU Langone Health, New York, NY, USA

**Keywords:** osteochondral defect, osteochondral lesion, surgery, surgical treatment, complication

## Abstract

**Objective:**

The primary aim was to determine and compare the complication rate of different surgical treatment options for osteochondral lesions of the talus (OLTs). The secondary aim was to analyze and compare the severity and types of complications.

**Design:**

A literature search was performed in MEDLINE (PubMed), EMBASE (Ovid), and the Cochrane Library. Methodological quality was assessed using the Methodological Index for Non-Randomized Studies (MINORS). Primary outcome was the complication rate per surgical treatment option. Secondary outcomes included the severity (using the Modified Clavien-Dindo-Sink Complication Classification System for Orthopedic Surgery) and types of complications. The primary outcome, the severity, and the sub-analyses were analyzed using a random effects model. A moderator test for subgroup-analysis was used to determine differences. The types of complications were presented as rates.

**Results:**

In all, 178 articles from the literature search were included for analysis, comprising 6,962 OLTs with a pooled mean age of 35.5 years and follow-up of 46.3 months. Methodological quality was fair. The overall complication rate was 5% (4%-6%; treatment group effect, *P* = 0.0015). Analysis resulted in rates from 3% (2%-4%) for matrix-assisted bone marrow stimulation to 15% (5%-35%) for metal implants. Nerve injury was the most observed complication.

**Conclusions:**

In 1 out of 20 patients treated surgically for an OLT, a complication occurs. Metal implants have a significantly higher complication rate compared with other treatment modalities. No life-threatening complications were reported.

## Introduction

Osteochondral lesions of the talus (OLTs) are lesions that affect the articular cartilage and the subchondral bone. Often, this arises after a traumatic event, such as an ankle sprain or fracture.^1,2^ Symptomatic OLTs often have a severe impact on the quality of life (QoL) of patients.^3^ To improve QoL, patients are treated, either non-operatively or surgically. In a stepped care treatment protocol, non-operative treatment is the first step, which has been shown being somewhat effective.^4-8^ However, if this is not successful, then surgical treatment may be considered necessary. The optimal surgical treatment is determined on the patients’ preferences, lesion nature, localization, size, morphology, and fixability of a fragment.^9^ A wide variety of surgical treatment options exist, including bone marrow stimulation (BMS) therapies, retrograde drilling, fixation, matrix-assisted BMS, cartilage implantation, osteo(chondral) autograft transplantation therapies, osteo(chondral) allograft transplantation therapies, and metal implants.^10-17^ In small lesions (defect size < 10 mm in any of the dimensions), fixation and BMS are the primary treatment options.^9^ For larger lesions, retrograde drilling and osteo(chondral) transplantation therapies are more often used.^9^

Every different surgical procedure comes with its own risks and complications. Complication characteristics are an important factor in the evidence-based shared decision-making process.^18^ Besides complications, the efficacy and costs of a treatment are also of importance. Recent research has shown that no superior treatment for primary and secondary lesions exists.^19,20^ Increased insight into the occurrence, severity, and types of complications in surgical treatment of OLTs is therefore of importance in future patients’ treatment decisions.

The primary aim of this study is to determine and compare the complication rate per surgical treatment option for OLTs. The secondary purpose is to analyze and compare the severity of complications and to analyze the types of complications per treatment option. The hypothesis of the present study is that surgical treatment of OLTs yields a low complication rate.

## Materials and Methods

The protocol of the present study was registered prospectively in the international prospective registry for systematic reviews PROSPERO^21^ (registration number CRD42018081490). The Preferred Reporting Items for Systematic Reviews and Meta-Analyses (PRISMA) statement was followed as a guideline for this study.^22^

### Search Strategy

Studies from 1996 until September 2021 from MEDLINE (PubMed), EMBASE (Ovid), and the Cochrane Library were identified. The publication filter from 1996 was applied due to the emerging of arthroscopy in the ankle. The full search strategy can be found in the Supplementary Appendix. Backward citation chaining was performed to find any additional eligible articles.

### Eligibility Criteria and Study Selection

All clinical studies that investigated surgical treatment of OLTs were eligible for inclusion. The full text needed to be available in English, French, Spanish, German, or Dutch. No restrictions regarding patient demographics were applied. A study needed to analyze a minimum of 5 OLTs on a minimum follow-up of 6 months. Review, cadaver, and animal studies were excluded. In case there was overlap between studies, the study with the highest number of patients was included. If multiple treatment modalities were used in an eligible study, the authors of the present study needed to be able to extract the data per treatment option to include the study. The title and abstract was screened by two researchers (J.H. and J.D.). This was performed using the Rayyan web-tool, which makes it more convenient for researchers to screen articles.^23^

### Methodological Quality

Methodological quality was assessed by two independent reviewers (J.H. and J.D.) using the Methodological Index for Non-Randomized Studies (MINORS).^24^

### Data Extraction

Data extraction was performed by two reviewers (J.H. and J.D.) using a standardized extraction form, specially designed for the present study, and tested before use. Data on study and patient characteristics were collected. Study characteristics include author, title, year of publication, level of evidence, treatments used, and follow-up duration. Patient characteristics are gender, age, laterality, and complications.

The definition of a complication, used in this study, is derived from the definition of Sokol and Wilson,^25^ and is as follows: “any undesirable, unintended, and direct result of an operation affecting the patient.” Any complication related to the success rate of the treatment, for example, graft non-union or other graft-related failures, was excluded from the analysis. In addition, secondary surgical procedures were also not scored as a complication. This was done to prevent the double-counting of complications for which subsequent surgical treatment was needed (e.g., complication and subsequent needed surgery counts one complication).^26^ Also hardware removal was not considered a complication.^27-29^

The severity of the complications was assessed with the help of the Modified Clavien-Dindo-Sink Complication Classification System for Orthopedic Surgery (**Table 1**).^30^ This classification was used to avoid subjective terms as “major” and “minor” and to improve the interrater and intrarater reliability.^30^ To compare the different surgical treatment options, treatment groups based on treatment modalities were defined. The treatment modalities are BMS therapies, retrograde drilling, fixation, matrix-assisted BMS, cartilage implantation, osteo(chondral) autograft transplantation therapies, osteo(chondral) allograft transplantation therapies, and metal implants (**Table 2**). The different types of complications were also extracted, to assess which type of complications occurs per treatment option.

**Table 1.** Modified Clavien-Dindo-Sink Complication Classification System for Orthopedic Surgery.

Grade	Definition
I	A complication that does not result in deviation from routine follow-up in the postoperative period and has minimal clinical relevance and requires minimal treatment (e.g., antiemetics, antipyretics, analgesics, diuretics, electrolytes, antibiotics, and physiotherapy) or no treatment.
II	A deviation from the normal postoperative course (including unplanned clinic/office visits) that requires outpatient treatment, either pharmacological or close monitoring as an outpatient.
III	A complication that is treatable but requires surgical, endoscopic, or interventional radiology procedure(s), or an unplanned hospital readmission.
IVa	A complication that is life- or limb-threatening, and/or requires ICU admission, a complication with potential for permanent disability but treatable, a complication that may require organ/joint resection/replacement. No long-term disability.
IVb	A complication that is life- or limb-threatening, and/or requires ICU admission, a complication that is not treatable, a complication that requires organ/joint resection/replacement or salvage surgery. With long-term disability.
V	Death.

Adopted from Dodwell *et al.*^30^

ICU = intensive care unit.

**Table 2.** Treatment Modalities.

Treatment Option	Examples
Bone marrow stimulation therapies	Bone marrow stimulation, debridement, microfracture
Retrograde drilling	Retrograde drilling
Fixation	Lift, drill, fill, fix
Matrix-assisted bone marrow stimulation	Autologous chondrocyte inducing chondrogenesis, matrix-associated stem cell transplantation, autologous matrix-induced chondrogenesis, bone marrow–derived stem cell transplantation
Cartilage implantation	Autologous chondrocyte implantation, matrix-induced autologous chondrocyte implantation, particulated juvenile cartilage allograft transplantation
Osteo(chondral) autograft transplantation therapies	Osteochondral autograft transfer system, autologous osteochondral transplantation
Osteo(chondral) allograft transplantation therapies	Allogenic osteochondral transplantation
Metal implants	Implant therapies

### Statistical Analysis

The primary outcome is the complication rate, which is defined as the proportion of complications to the total number of lesions. This was then pooled per surgical treatment option (i.e., BMS therapies, retrograde drilling, fixation, matrix-assisted BMS, cartilage implantation, osteo(chondral) autograft transplantation therapies, osteo(chondral) allograft transplantation therapies, and metal implants) with a random effects model, weighted by inverse variance. *T*^2^, a measure of heterogeneity, was determined with the use of the DerSimonian and Laird^31^ estimator, and the corresponding confidence interval (CI) was calculated using the Jackson and Bowden^32^ method. A logit transformation was applied. The 95% CIs for individual studies were calculated using the Clopper-Pearson^33^ interval method. Difference in complication rates within treatment modalities were compared using a moderator test for subgroup-analysis with α = 0.05.^34^ If a significant effect among all treatments was found, *post hoc* moderator tests were used to assess difference between single treatment modalities.

The distribution of grades per treatment option and the types of complications were presented as a rate which is the proportion of the total complications per treatment option. The different types of complications were presented by means of a top 5 per treatment option.

All analyses were performed in STATA 15 (StateCorp LP, College Station, TX) and R version 4.0.2 (RStudio, Boston, MA) with meta package.^35^

## Results

### Article Selection

**Figure 1.**
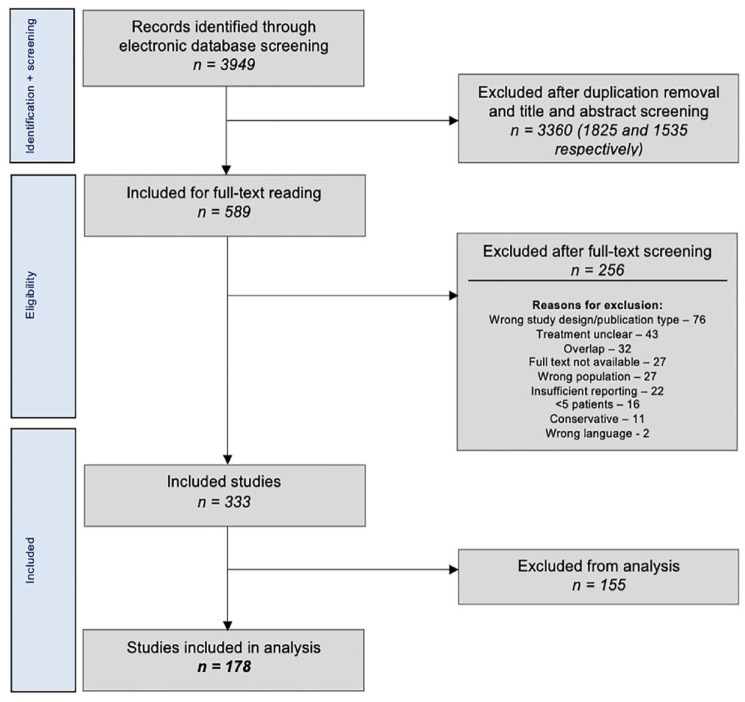
Preferred Reporting Items for Systematic Reviews and Meta-Analyses flowchart of study selection.

The literature search yielded 3,949 articles, of which 333 met the inclusion criteria (**Fig. 1**). A total of 155 studies comprising 6,261 lesions could not be included in the analysis as they did not (well) report complications. The distribution of the lesions among the 155 studies can be viewed in **Table 3**. In all, 178 studies remained for analysis in the present study.

**Table 3.** Characteristics Excluded Studies Due to Underreporting/Unclear Reporting of Complications.

Treatment Option	Lesions *n*
Overall	6,261
Bone marrow stimulation therapies	2,916
Retrograde drilling	253
Fixation	181
Matrix-assisted bone marrow stimulation	1,031
Cartilage implantation	493
Osteo(chondral) autograft transplantation therapies	1,068
Osteo(chondral) allograft transplantation therapies	236
Metal implants	41
Other	42

### Evaluation of the Characteristics of Included Studies

Overall, the 178 studies included 6,921 patients with 6,962 lesions (41 bilateral patients [0.6% of all patients]). The pooled mean age was 35.5 years (range of means: 10.4-58), and the pooled mean follow-up was 46.3 months (range of means: 6-411.6 months).

### Methodological Quality

Scoring the methodological quality of the 134 non-comparative studies resulted in an average score of 10.1 (range: 5-13) out of 16 points.^11,14,15,17,27-29,36-162^ The 44 comparative studies had an average score of 17.8 (range: 13-22) out of 24 points.^163-206^ The MINORS scores per individual study can be found in the Supplementary Appendix.

### Primary Outcome

In total, 225 complications were reported. The overall complication rate was 5% (95% CI: 4%-6%; treatment group effect *Q* = 25.05, *P* = 0.0015), ranging per treatment option from 3% (95% CI: 2%-4%) for matrix-assisted BMS to 15% (95% CI: 5%-35%) for metal implants. The moderator test for subgroup-analysis between all treatment modalities showed a significant effect of treatment option (*Q* = 25.05, *P* = 0.015). Metal implants have a higher complication rate compared with BMS therapies (*Q* = 5.27, *P* = 0.0217), compared with retrograde drilling (*Q* = 3.86, *P* = 0.0493), compared with fixation (*Q* = 4.56, *P* = 0.0328), compared with matrix-assisted BMS (*Q* = 9.29, *P* = 0.0023), and compared with cartilage implantation (*Q* = 3.95, *P* = 0.0468). In addition, differences between matrix-assisted BMS and osteo(chondral) autograft transplantation therapies (3% [95% CI: 2%-5%] versus 7% [95% CI: 5%-10%], *Q* = 16.86, *P* ≤ 0.0001) and between matrix-assisted BMS and osteo(chondral) allograft transplantation therapies (3% [95% CI: 2%-5%] versus 8% [95% CI: 4%-16%], *Q* = 7.66, *P* = 0.0056) were found. The overall complication rate and the complication rate per treatment option can be viewed in **Table 4**.

**Table 4.** Overall Complication Rate per Treatment Option.

Treatment Option	Studies *n*	Lesions *n*	Pooled Mean Age (Years)	Pooled Mean Follow-Up (Months)	Complication Rate % (95% Confidence Interval)
Overall	178	6,962	35.5	46.3	5 (4-6)
Bone marrow stimulation therapies^a^	67	2,926	36.7	48.7	4 (3-6)^a^
Retrograde drilling^b^	14	289	32.6	39.2	5 (3-8)^b^
Fixation^c^	8	179	22.3	35.5	3 (1-8)^c^
Matrix-assisted bone marrow stimulation^d^	31	1,072	16.8	14.7	3 (2-4)^d^
Cartilage implantation^e^	22	479	32.8	47.5	5 (3-8)^e^
Osteo(chondral) autograft transplantation therapies^f^	61	1,639	34.7	54.2	8 (6-10)^f^
Osteo(chondral) allograft transplantation therapies^g^	15	288	43.1	30.8	8 (4-14)^g^
Metal implants^h^	3	80	41.7	54.4	15 (5-35)^h^
Other^i^	1	10	43.3	12	5 (0-45)^i^

a Significantly different from osteo(chondral) autograft transplantation therapies (*Q* = 7.55, *P* = 0.0060) and metal implants (*Q* = 5.27, *P* = 0.0217).^43,52,59,61-63,66,81,92,93,109,113,117,121,123,125,131,135,141,143,146,149,150,153,158,164,165,167,168,171,173,176,177,179-183,187,190,192,195-200,202,204-206^

b Significantly different from metal implants (*Q* = 3.86, *P* = 0.0493).^11,42,53,56,59,116,118,137,170,177,194,196^

c Significantly different from metal implants (*Q* = 4.56, *P* = 0.0328).^85,102,104,119,134,138,185,186^

d Significantly different from osteo(chondral) autograft transplantation therapies (*Q* = 16.86, *P* < 0.0001), osteo(chondral) allograft transplantation therapies (*Q* = 7.66, *P* = 0.0056), and metal implants (*Q* = 9.29, *P* = 0.0023).^29,38,40,49,58,65,72,77,82,94,98,101,130,139,140,142,144,145,155,157,162,166,169,181,189,192,195,201,204^

e Significantly different from metal implants (*Q* = 3.95, *P* = 0.0468).^14,41,45,46,51,64,76,78-80,86,99,103,107,112,114,115,124,133,147,179^

f Multiple studies: ^15,36,39,47,48,50,60,68,70,71,74,75,83,84,87-90,95-97,100,105,106,108,110,120,126,128,129,132,136,138,148,151,152,154,156,159-161,163,171-175,178,184,188,191,193,194,197,203^.

g Multiple studies: ^17,37,44,54,55,57,67,73,91,111,122,152,163,191,206^.

h Multiple studies: ^27,28,69^.

i Study: ^127^.

### Secondary Outcomes

#### Severity of complications

No Grade IV and Grade V complications were found. Most of the complications were Grade I complications with an overall rate of 4% (95% CI: 3%-4%; difference between all treatment options, *Q* = 13.51, *P* = 0.0955). The rates for Grade II and Grade III are 3% (95% CI: 3%-4%; difference between all treatment options, *Q* = 12.88, *P* = 0.1160) and 3% (95% CI: 2%-3%; difference between all treatment options, *Q* = 8.44, *P* = 0.3917), respectively. The distribution of the severity of the complications that arise per treatment option can be seen in **Table 5**. The proportion of Grade I and Grade II (complications without the need of a hospital readmission or intervention) of all complications is 84%.

**Table 5.** Overall Complication Rate Separated in Severity.

Treatment Option	Grade I^a^		Grade II^a^		Grade III^a^	
	% (95% CI)	Proportion	% (95% CI)	Proportion	% (95% CI)	Proportion
Overall	4 (3-4)	51	3 (3-4)	33	3 (2-3)	16
Bone marrow stimulation therapies	3 (2-4)	48	2 (2-3)	30	2 (2-3)	22
Retrograde drilling	4 (2-7)	50	3 (2-7)	33	3 (1-6)	17
Fixation	3 (1-7)	50	3 (1-7)	N/A	3 (1-8)	50
Matrix-assisted bone marrow stimulation	2(1-3)	22	2 (1-3)	67	2 (1-3)	11
Cartilage implantation	4 (2-6)	44	3 (2-6)	44	3 (1-5)	11
Osteo(chondral) autograft transplantation therapies	6 (4-8)	62	4 (3-5)	28	3 (2-4)	10
Osteo(chondral) allograft transplantation therapies	3 (2-7)	13	6 (3-11)	47	5 (3-9)	40
Metal implants	7 (2-28)	50	7 (3-15)	42	3 (1-13)	8
Other	5 (0-45)	N/A	5 (0-45)	N/A	5 (0-45)	N/A

CI = confidence interval; N/A = not applicable.

a Severity based on the grades of Modified Clavien-Dindo-Sink Complication Classification System for Orthopedic Surgery. Rates presented as percentage of the overall complication rate of the respective treatment option, and the proportion of the grade of all complications overall and per treatment option. No significant differences were found.

#### Type of complications

The proportions of all complications for the most occurring complications per treatment option can be viewed in **Table 6**. In 5 treatment modalities (i.e., BMS therapies, retrograde drilling, fixation, cartilage implantation, and metal implants), nerve injury, either temporary or duration unknown, was the most frequently reported complication (range of the proportions of the total complications per treatment option: 22%-50%). For matrix-assisted BMS, superficial infection was the most occurring type of complication (56%), for osteochondral autograft transplantation therapies, it is donor site morbidity (41%), and deep venous thrombosis (DVT) for osteo(chondral) allograft transfer (27%).

**Table 6.** Most Occurring Complications per Treatment Option.

Treatment Option	Most Occurring		Second Most Occurring		Third Most Occurring		Fourth Most Occurring		Fifth Most Occurring	
	Complication	%	Complication	%	Complication	%	Complication	%	Complication	%
Bone marrow stimulation therapies	Nerve injury (dur. unk.)	22	Superficial infection	12	DVT	12	Nerve injury (temporary)	10	Hematoma	7
Retrograde drilling	Nerve injury (dur. unk.)	50	Arterial bleeding	17	Delayed wound healing	17	Extensor hallucis longus weakness	17	N/A	
Fixation	Nerve injury (temporary)	50	Screw loosening	50	N/A		N/A		N/A	
Matrix-assisted bone marrow stimulation	Superficial infection	56	Arthrofibrosis	11	Delayed union of osteotomy site	11	Nerve injury (temporary)	11	Donor site morbidity	11
Cartilage implantation	Nerve injury (temporary)	44	Delayed union of osteotomy site	22	Non-union of osteotomy site	11	Skin necrosis	11	Superficial infection	11
Osteo(chondral) autograft transplantation therapies	Donor site morbidity	41	Superficial infection	13	Nerve injury (temporary)	6	Nerve injury (dur. unk.)	3	Delayed union of osteotomy site	3
Osteo(chondral) allograft transplantation therapies^a^	DVT	27	Delayed union of osteotomy site	20	Plantar fasciitis	13	Arthrofibrosis	7	Cellulitis	7
Metal implants	Nerve injury (dur. unk.)	50	Wound dehiscence	25	Superficial infection	17	DVT	8	N/A	
Other	N/A		N/A		N/A		N/A		N/A	

Rates are the proportions from the total amount complications per treatment option. DVT = deep venous thrombosis; N/A = not applicable; dur. unk. = duration unknown.

a Nerve injury (duration unknown) and non-union both also have rates of 7% in this treatment option.

## Discussion

The main finding of the present study is that there is a 5% risk of a complication with surgical treatment of an OLT. Significant differences between treatment modalities exist, with metal implants having a significantly higher complication rate than BMS therapies, retrograde drilling, fixation, matrix-assisted BMS, and cartilage implantation. BMS therapies have a significantly lower complication than osteo(chondral) autograft transplantation therapies, and it has also been shown that matrix-assisted BMS results in significantly lower complication rates than osteo(chondral) autograft- and allograft transplantation therapies.

### Primary Outcome

There are multiple factors reported in the literature that affect the complication rate of OLT surgeries, such as operative time and the experience of the surgeon.^207,208^ The latter is of interest in the context of the present study as ankle arthroscopy is regarded as a challenging procedure.^209^ Accordingly, this is of importance regarding the possible complications, considering damaging the peroneal nerve near the lateral portal in anterior ankle arthroscopy.^210,211^

Metal implants have a significantly higher complication rate compared with retrograde drilling, fixation, matrix-assisted BMS, and cartilage implantation. This may be due to several reasons. First, for the implantation of a metal implant, an open procedure with an osteotomy is often needed. This primarily, already, introduces a higher risk for potential infections, nerve injury, and other types of complications. This is because a longer incision is often needed which could damage nerve branches and introduce a greater *porte d’entrée*. This clarifies why in the analysis of the type of complications, the present study also found nerve damage and superficial infections to be more frequent in metal implants. Second, implantation of a metal resurfacing inlay implant may cause degenerative changes to the distal tibia.^27^ The literature has found rates from 10% to 60%.^16,69^ Third, the learning curve of this procedure is fairly long.^69,212^ This is due in part to the fact that the depth of the implantation is of importance, due to the deformity of the talar cartilage during load.^213,214^ As mentioned above, the experience of a surgeon is of vast importance for the complication rate. Given the potential limited experience with these implants, may cause higher complication rates. In addition, it must be noted that metal implantation often is used as a salvage procedure and that may influence the complication rate. There is, however, an intrinsic relationship between the indication and the treatment technique and the other treatment groups also include a subset of secondary patients.

### Secondary Outcomes

The severity of the complications was assessed with the use of the Modified Clavien-Dindo-Sink Complication Classification System for Orthopedic Surgery.^30^ No Grade IV (a and b) and V complications were found. Grade I, II, and III complications have overall rates of 4%, 3%, and 3%, respectively. No significant difference was found for either grade.

In 5 treatment modalities (i.e., BMS therapies, retrograde drilling, fixation, cartilage implantation, and metal implants), nerve injury (temporary and duration unknown) was the most occurring complication. In the other treatment modalities, superficial infection (matrix-assisted BMS), donor site morbidity (osteo(chondral) autograft transplantation therapies) and DVT (osteo(chondral) allograft transplantation therapies) occurred the most times. All types of complications found in the present study are treatable and not life-threatening. It should be mentioned that the term nerve injury comprises complications from dysesthesia, neuralgia, paraesthesia, and altered sensation.

### Defining the Term “Complication”

It is of critical importance in the analysis of complications to determine the definition of the term “complication.” Unfortunately, there is currently no widely accepted uniform definition for this term available, resulting in high heterogeneity in the reporting of complications in the literature. Factors related to the treatment failure were explicitly not considered as complications by the authors. This includes graft-related complications, such as failure to incorporate and reject a graft, as well as issues like persistent pain and no improvement of function. The removal of possible implanted hardware was also not included. Many studies removed screws from an osteotomy (reported rates of 17%-48% in 9 studies) which could also be for reasons other than, for example, irritation over the screw head.^15,27-29,83,87,122,133,179^ As a result of the high heterogeneity in the definition of a complication, some studies report possible purely radiological findings, such as bone marrow edema and subchondral cyst formation, as a complication.^112,176^ These findings were not counted as a complication in the present study, as they may not have any clinical consequences.

To improve this troublesome situation, the present study recommends that a proper definition should be established for complications in the treatment of OLTs. A definition could be drafted on a consensus meeting.

### Methodological Considerations

The methodological quality of the included articles can be considered fair, as shown by the MINORS score. Many studies were of a non-comparative design and only 7 randomized controlled trials (RCTs) were included. This may be of influence for the results, as a higher methodological quality is possibly closer to the actual complication rate of procedure. Although it was our intention to perform a sub-analysis on the complication rate and severity of primary OLTs versus non-primary OLTs, it must be stated that this was not possible due to the heterogeneous patient groups and limited reporting.

In the literature search, only articles from 1996 were searched. This filter was applied because the technique of ankle arthroscopy only really emerged since this year. It became more and more normal to use this surgical technique. This is due to technological advances and increased clarity about (the applicability of) the technique.^215-217^

In the methodology of the present study, it was decided not to contact authors of articles in case of any doubt. There are, however, a high number of articles included, and therefore, the authors expect that this will have little impact on the results. In all, 137 studies (46% of the included articles) were excluded for the analysis because the complications were not (clearly) described.

Among the included studies, some were of retrospective nature, which may affect the complication rates. In effect, there may be underreporting due to selection, detection, and recall biases. Therefore, the complication rates found in the present study may be in reality higher. A 2-fold difference has been found in the literature.^218^

### Clinical Relevance and Future Perspectives

This study presents the complication rate per treatment option for OLTs and significant differences between treatment modalities. The complication rate and severity, in combination with the clinical efficacy and the costs, are one of the most important factors in the treatment selection. The clinical efficacy has already been recently reviewed, finding no clearly superior treatment for either primary or secondary lesions.^19,20^ Thus, to justify the treatment choice, mere complication and cost factors remain. The insight of this study that surgical treatment of OLTs can be considered safe and that there are differences between treatment modalities may be of importance in the consulting room. It can, thus, help clinicians and patients in the evidence-based shared decision-making process and can also manage postoperative expectations of clinicians and patients.

The present study was not able to analyze the surgery duration. This was often not reported and in the case of reporting, then it was not clear. For example, it was often not known to which treatment group it applied and what the start and end points of the measurement were. Surgery duration, however, has been identified as an independent risk factor for postoperative complications in orthopedic surgery.^219-226^ Further research on risk factors, specific for this type of surgery, needs to be done in the future.

## Conclusion

In 1 out of 20 patients treated surgically for an OLT, a complication occurs. Metal implants have a significantly higher complication rate than BMS therapies, retrograde drilling, fixation, matrix-assisted BMS, and cartilage implantation. BMS and matrix-assisted BMS have significantly lower complication rates compared with osteo(chondral) autograft transplantation therapies. In addition, matrix-assisted BMS is also significantly lower than osteo(chondral) allograft transplantation therapies. No life-threatening complications were reported. Nerve injuries were the most frequently observed type of complications for BMS therapies, retrograde drilling, fixation, cartilage implantation, and metal implants.

## Supplemental Material

sj-docx-1-car-10.1177_19476035231154746 – Supplemental material for The Frequency and Severity of Complications in Surgical Treatment of Osteochondral Lesions of the Talus: A Systematic Review and Meta-Analysis of 6,962 Lesions Click here for additional data file. Supplemental material, sj-docx-1-car-10.1177_19476035231154746 for The Frequency and Severity of Complications in Surgical Treatment of Osteochondral Lesions of the Talus: A Systematic Review and Meta-Analysis of 6,962 Lesions by Julian J. Hollander, Jari Dahmen, Kaj S. Emanuel, Sjoerd A.S. Stufkens, John G. Kennedy and Gino M.M.J. Kerkhoffs in CARTILAGE

sj-jpg-2-car-10.1177_19476035231154746 – Supplemental material for The Frequency and Severity of Complications in Surgical Treatment of Osteochondral Lesions of the Talus: A Systematic Review and Meta-Analysis of 6,962 Lesions Click here for additional data file. Supplemental material, sj-jpg-2-car-10.1177_19476035231154746 for The Frequency and Severity of Complications in Surgical Treatment of Osteochondral Lesions of the Talus: A Systematic Review and Meta-Analysis of 6,962 Lesions by Julian J. Hollander, Jari Dahmen, Kaj S. Emanuel, Sjoerd A.S. Stufkens, John G. Kennedy and Gino M.M.J. Kerkhoffs in CARTILAGE

sj-jpg-3-car-10.1177_19476035231154746 – Supplemental material for The Frequency and Severity of Complications in Surgical Treatment of Osteochondral Lesions of the Talus: A Systematic Review and Meta-Analysis of 6,962 Lesions Click here for additional data file. Supplemental material, sj-jpg-3-car-10.1177_19476035231154746 for The Frequency and Severity of Complications in Surgical Treatment of Osteochondral Lesions of the Talus: A Systematic Review and Meta-Analysis of 6,962 Lesions by Julian J. Hollander, Jari Dahmen, Kaj S. Emanuel, Sjoerd A.S. Stufkens, John G. Kennedy and Gino M.M.J. Kerkhoffs in CARTILAGE

sj-jpg-4-car-10.1177_19476035231154746 – Supplemental material for The Frequency and Severity of Complications in Surgical Treatment of Osteochondral Lesions of the Talus: A Systematic Review and Meta-Analysis of 6,962 Lesions Click here for additional data file. Supplemental material, sj-jpg-4-car-10.1177_19476035231154746 for The Frequency and Severity of Complications in Surgical Treatment of Osteochondral Lesions of the Talus: A Systematic Review and Meta-Analysis of 6,962 Lesions by Julian J. Hollander, Jari Dahmen, Kaj S. Emanuel, Sjoerd A.S. Stufkens, John G. Kennedy and Gino M.M.J. Kerkhoffs in CARTILAGE

sj-jpg-5-car-10.1177_19476035231154746 – Supplemental material for The Frequency and Severity of Complications in Surgical Treatment of Osteochondral Lesions of the Talus: A Systematic Review and Meta-Analysis of 6,962 Lesions Click here for additional data file. Supplemental material, sj-jpg-5-car-10.1177_19476035231154746 for The Frequency and Severity of Complications in Surgical Treatment of Osteochondral Lesions of the Talus: A Systematic Review and Meta-Analysis of 6,962 Lesions by Julian J. Hollander, Jari Dahmen, Kaj S. Emanuel, Sjoerd A.S. Stufkens, John G. Kennedy and Gino M.M.J. Kerkhoffs in CARTILAGE
