# Supplementary material for: The Frequency and Severity of Complications in Surgical Treatment of Osteochondral Lesions of the Talus: A Systematic Review and Meta-Analysis of 6,962 Lesions
Source: Cartilage. 2023 Mar 9;14(2):180–97. doi: 10.1177/19476035231154746 (PMC10416205; doi:10.1177/19476035231154746)
Supplement: sj-docx-1-car-10.1177_19476035231154746 – Supplemental material for The Frequency and Severity of Complications in Surgical Treatment of Osteochondral Lesions of the Talus: A Systematic Review and Meta-Analysis of 6,962 Lesions [file sj-docx-1-car-10.1177_19476035231154746.docx]

APPENDIX

*Appendix 1: Search Strategy*

| **#** | **MEDLINE (PubMed)** | **EMBASE (Ovid)** | **The Cochrane Library** |
| --- | --- | --- | --- |
| 1 | "Osteochondritis Dissecans"[MeSH] | osteochondritis dissecans/ or (osteochondritis dissecans or osteochondrosis dissecans or osteochondrolysis or OCD or OLT).ti,ab,kw. or ((osteochondral or chondral or osteochondral or transchondral or cartilage*) adj3 (defect* or lesion*)).ti,ab,kw. | MeSH descriptor: [Osteochondritis Dissecans] explode all trees |
| 2 | osteochondritis dissecans[tiab] OR osteochondrosis dissecans[tiab] OR osteochondrolysis[tiab] OR OCD[tiab] OR OLT[tiab] | (osteochondritis dissecans/ or (osteochondritis dissecans or osteochondrosis dissecans or osteochondrolysis or OCD or OLT).ti,ab,kw. or ((osteochondral or chondral or osteochondral or transchondral or cartilage*) adj3 (defect* or lesion*)).ti,ab,kw.) and (talus/ or (talus or talar* or ankle).ti,ab,kw.) | osteochondritis dissecans or osteochondrosis dissecans or osteochondrolysis or OCD or OLT:ti,ab,kw (Word variations have been searched) |
| 3 | (osteochondral[tiab] OR chondral[tiab] OR transchondral[tiab] OR cartilage*[tiab]) AND (defect*[tiab] OR lesion*[tiab]) | limit 2 to yr&#61;"1996-Current" | (osteochondral or chondral or transchondral or cartilage*) and (defect* or lesion*):ti,ab,kw (Word variations have been searched) |
| 4 | #1 OR #2 OR #3 |  | #1 or #2 or #3 |
| 5 | “Talus”[MeSH] |  | MeSH descriptor: [Talus] explode all trees |
| 6 | talus[tiab] OR talar*[tiab] OR ankle[tiab] |  | talus or talar* or ankle:ti,ab,kw (Word variations have been searched) |
| 7 | #5 OR #6 |  | #5 or #6 |
| 8 | #4 AND #7 |  | #4 and #7, Publication Year from 1996 to 2016, in Cochrane Reviews (Reviews and Protocols), Other Reviews and Trials |

*Appendix 2: MINORS Scores*

| MINORS | | | | | | | | | Additional criteria for comparative studies | | | |  |
| --- | --- | --- | --- | --- | --- | --- | --- | --- | --- | --- | --- | --- | --- |
| Study | A clearly stated aim | Inclusion of consecutive patients | Prospective collection of data | Endpoint appropiate to the aim of the study | Unbiased assesment of the study endpoint | Follow up period appropiate to the aim of the study | Lost of follow up less than 5% | Prospective calculation of study size | An adequate control group | Contampory group | Baseline equivalent of groups | Adequate statistical analysis | Total |
| Adams Jr, 2011^17^ | 2 | 2 | 1 | 2 | 1 | 2 | 2 | 0 |  |  |  |  | 12/16 |
| Ahmad, 2016^163^ | 2 | 2 | 2 | 2 | 1 | 2 | 2 | 0 | 2 | 2 | 1 | 1 | 19/24 |
| Ahmad, 2017^37^ | 2 | 2 | 2 | 2 | 1 | 2 | 2 | 0 |  |  |  |  | 13/16 |
| Akmese, 2020^38^ | 2 | 1 | 0 | 2 | 1 | 2 | 2 | 0 |  |  |  |  | 10/16 |
| Albano, 2017^40^ | 2 | 1 | 1 | 2 | 1 | 2 | 2 | 0 |  |  |  |  | 11/16 |
| Allahabadi, 2021^164^ | 2 | 1 | 1 | 2 | 1 | 2 | 2 | 0 | 2 | 2 | 2 | 2 | 19/24 |
| Al-Shaikh, 2002^39^ | 0 | 1 | 0 | 0 | 1 | 2 | 2 | 0 |  |  |  |  | 6/16 |
| Anders, 2012^41^ | 1 | 2 | 1 | 1 | 1 | 2 | 2 | 0 |  |  |  |  | 10/16 |
| Anders, 2012^42^ | 1 | 2 | 1 | 1 | 1 | 2 | 2 | 0 |  |  |  |  | 10/16 |
| Angthong, 2013^43^ | 2 | 2 | 0 | 2 | 2 | 2 | 2 | 0 |  |  |  |  | 12/16 |
| Arauz, 2017^44^ | 2 | 2 | 1 | 2 | 1 | 2 | 2 | 0 |  |  |  |  | 12/16 |
| Arican, 2019^165^ | 1 | 2 | 1 | 2 | 1 | 2 | 2 | 0 | 2 | 2 | 2 | 2 | 19/24 |
| Assenmacher, 2001^36^ | 2 | 2 | 1 | 2 | 1 | 2 | 2 | 0 |  |  |  |  | 12/16 |
| Aurich, 2011^45^ | 2 | 1 | 1 | 2 | 1 | 2 | 2 | 0 |  |  |  |  | 11/16 |
| Bai, 2020^46^ | 2 | 2 | 1 | 2 | 1 | 2 | 2 | 0 |  |  |  |  | 12/16 |
| Basal, 2020^47^ | 1 | 2 | 2 | 2 | 1 | 2 | 2 | 0 |  |  |  |  | 12/16 |
| Batista, 2020^153^ | 2 | 2 | 2 | 2 | 1 | 2 | 2 | 0 |  |  |  |  | 13/16 |
| Battaglia, 2011^48^ | 2 | 1 | 1 | 2 | 1 | 2 | 2 | 0 |  |  |  |  | 11/16 |
| Baumfeld, 2018^49^ | 2 | 2 | 1 | 2 | 1 | 2 | 2 | 0 |  |  |  |  | 12/16 |
| Baumgartner, 2006^50^ | 1 | 2 | 1 | 1 | 1 | 2 | 2 | 0 |  |  |  |  | 10/16 |
| Baums, 2006^51^ | 1 | 1 | 2 | 1 | 1 | 2 | 2 | 0 |  |  |  |  | 10/16 |
| Becher, 2005^52^ | 2 | 1 | 2 | 2 | 1 | 2 | 2 | 0 |  |  |  |  | 12/16 |
| Beck, 2016^53^ | 1 | 1 | 2 | 1 | 1 | 2 | 2 | 0 |  |  |  |  | 10/16 |
| Berlet, 2011^54^ | 1 | 2 | 2 | 1 | 1 | 2 | 2 | 0 |  |  |  |  | 11/16 |
| Bleazey, 2012^55^ | 0 | 1 | 1 | 0 | 1 | 2 | 2 | 0 |  |  |  |  | 7/16 |
| Bohnsack, 2003^56^ | 2 | 1 | 1 | 2 | 1 | 2 | 2 | 0 |  |  |  |  | 11/16 |
| Brigido, 2014^57^ | 2 | 2 | 1 | 2 | 1 | 2 | 2 | 0 |  |  |  |  | 12/16 |
| Buda, 2013^58^ | 2 | 2 | 2 | 2 | 1 | 2 | 2 | 0 |  |  |  |  | 13/16 |
| Cadossi, 2014^166^ | 2 | 2 | 2 | 2 | 2 | 2 | 2 | 0 | 2 | 2 | 1 | 2 | 21/24 |
| Camurcu, 2020^167^ | 2 | 2 | 1 | 2 | 1 | 2 | 2 | 0 | 2 | 2 | 2 | 2 | 20/24 |
| Carlson, 2020^59^ | 2 | 1 | 1 | 2 | 1 | 2 | 2 | 0 |  |  |  |  | 11/16 |
| Chen, 2015^60^ | 1 | 0 | 2 | 1 | 1 | 2 | 2 | 0 |  |  |  |  | 9/16 |
| Choi, 2013^168^ | 2 | 2 | 1 | 2 | 1 | 2 | 2 | 0 | 2 | 2 | 1 | 2 | 19/24 |
| Chuckpaiwong, 2008^61^ | 2 | 2 | 2 | 2 | 1 | 2 | 2 | 0 |  |  |  |  | 13/16 |
| Cuttica, 2011^62^ | 2 | 2 | 0 | 2 | 1 | 2 | 2 | 0 |  |  |  |  | 11/16 |
| D’Ambrosi, 2017^169^ | 2 | 2 | 1 | 2 | 1 | 2 | 2 | 0 | 1 | 2 | 0 | 0 | 15/24 |
| Danilkowicz, 2022^199^ | 1 | 2 | 0 | 2 | 1 | 1 | 2 | 0 | 2 | 2 | 1 | 2 | 16/24 |
| De l’Escalopier, 2022^154^ | 2 | 2 | 0 | 2 | 1 | 2 | 2 | 0 |  |  |  |  | 11/16 |
| De Lima, 2011^63^ | 1 | 2 | 1 | 2 | 1 | 2 | 2 | 0 |  |  |  |  | 11/16 |
| DeSandis, 2018^64^ | 2 | 1 | 1 | 2 | 1 | 2 | 2 | 0 |  |  |  |  | 11/16 |
| Di Cave, 2017^65^ | 2 | 2 | 1 | 2 | 1 | 2 | 2 | 0 |  |  |  |  | 12/16 |
| Doral, 2012^66^ | 2 | 2 | 2 | 2 | 1 | 2 | 2 | 0 |  |  |  |  | 13/16 |
| Ebskov, 2020^27^ | 0 | 2 | 2 | 0 | 1 | 1 | 2 | 0 |  |  |  |  | 8/16 |
| El-Rashidy, 2011^67^ | 1 | 2 | 2 | 1 | 1 | 2 | 2 | 0 |  |  |  |  | 11/16 |
| El Sallakh, 2012^131^ | 2 | 2 | 1 | 2 | 1 | 2 | 2 | 0 |  |  |  |  | 12/16 |
| Emre, 2012^68^ | 1 | 2 | 1 | 2 | 0 | 2 | 2 | 0 |  |  |  |  | 10/16 |
| Ettinger, 2017^69^ | 1 | 2 | 0 | 1 | 0 | 2 | 2 | 0 |  |  |  |  | 8/16 |
| Flynn, 2016^70^ | 1 | 2 | 0 | 1 | 1 | 2 | 2 | 0 |  |  |  |  | 9/16 |
| Fraser, 2016^71^ | 1 | 1 | 0 | 1 | 1 | 2 | 2 | 0 |  |  |  |  | 8/16 |
| Galla, 2019^72^ | 2 | 2 | 1 | 2 | 2 | 2 | 2 | 0 |  |  |  |  | 13/16 |
| Galli, 2015^73^ | 2 | 1 | 2 | 2 | 1 | 2 | 2 | 0 |  |  |  |  | 12/16 |
| Gao, 2017^170^ | 2 | 2 | 1 | 2 | 1 | 2 | 2 | 0 | 2 | 2 | 2 | 2 | 20/24 |
| Gautier, 2002^74^ | 1 | 1 | 0 | 1 | 1 | 2 | 2 | 0 |  |  |  |  | 8/16 |
| Georgiannos, 2016^75^ | 1 | 2 | 0 | 1 | 0 | 2 | 2 | 0 |  |  |  |  | 8/16 |
| Geyer, 2022^155^ | 1 | 2 | 0 | 2 | 0 | 0 | 1 | 0 |  |  |  |  | 6/16 |
| Gianakos, 2022^200^ | 2 | 2 | 0 | 2 | 1 | 2 | 2 | 0 | 2 | 2 | 1 | 2 | 18/24 |
| Giannini, 2001^78^ | 0 | 1 | 0 | 1 | 1 | 2 | 2 | 0 |  |  |  |  | 7/16 |
| Giannini, 2005^79^ | 2 | 0 | 1 | 2 | 1 | 1 | 2 | 0 |  |  |  |  | 9/16 |
| Giannini, 2008^80^ | 2 | 2 | 1 | 2 | 1 | 0 | 2 | 0 |  |  |  |  | 10/16 |
| Giannini, 2009^76^ | 2 | 1 | 1 | 2 | 1 | 2 | 2 | 0 |  |  |  |  | 11/16 |
| Giannini, 2013^77^ | 1 | 2 | 1 | 1 | 1 | 2 | 2 | 0 |  |  |  |  | 10/16 |
| Giannini, 2014^14^ | 2 | 2 | 1 | 2 | 1 | 2 | 2 | 0 |  |  |  |  | 12/16 |
| Gobbi, 2006^171^ | 1 | 1 | 2 | 1 | 1 | 2 | 2 | 0 | 2 | 0 | 1 | 0 | 13/24 |
| Goh, 2015^81^ | 1 | 2 | 1 | 1 | 0 | 1 | 2 | 0 |  |  |  |  | 8/16 |
| Gu, 2017^82^ | 1 | 1 | 1 | 1 | 1 | 1 | 2 | 0 |  |  |  |  | 8/16 |
| Gül, 2015^83^ | 2 | 1 | 0 | 1 | 1 | 1 | 2 | 0 |  |  |  |  | 8/16 |
| Gül, 2016^172^ | 2 | 2 | 1 | 2 | 1 | 2 | 2 | 0 | 2 | 2 | 2 | 2 | 20/24 |
| Guney, 2016^173^ | 0 | 2 | 1 | 0 | 1 | 2 | 2 | 0 | 2 | 2 | 1 | 2 | 13/24 |
| Haasper, 2008^174^ | 1 | 2 | 1 | 2 | 0 | 2 | 2 | 0 | 2 | 2 | 0 | 0 | 14/24 |
| Haleem, 2014^175^ | 1 | 2 | 1 | 1 | 1 | 2 | 2 | 0 | 2 | 2 | 1 | 2 | 17/24 |
| Hangody, 2001^84^ | 0 | 1 | 0 | 0 | 1 | 2 | 2 | 0 |  |  |  |  | 6/16 |
| Hannon, 2016^176^ | 1 | 2 | 1 | 1 | 1 | 2 | 2 | 0 | 2 | 2 | 1 | 2 | 17/24 |
| Haraguchi, 2020^85^ | 2 | 2 | 1 | 2 | 1 | 2 | 2 | 0 |  |  |  |  | 12/16 |
| Heida, 2020^86^ | 1 | 2 | 1 | 1 | 1 | 2 | 2 | 0 |  |  |  |  | 10/16 |
| Hintermann, 2015^87^ | 1 | 1 | 2 | 1 | 1 | 2 | 2 | 0 |  |  |  |  | 10/16 |
| Hu, 2013^88^ | 2 | 2 | 1 | 2 | 1 | 2 | 2 | 0 |  |  |  |  | 12/16 |
| Hu, 2021^89^ | 2 | 1 | 0 | 2 | 1 | 2 | 2 | 0 |  |  |  |  | 10/16 |
| Hyer, 2008^11^ | 1 | 2 | 1 | 1 | 1 | 2 | 2 | 0 |  |  |  |  | 10/16 |
| Imhoff, 2011^90^ | 2 | 2 | 0 | 2 | 1 | 2 | 2 | 0 |  |  |  |  | 11/16 |
| Jackson, 2019^91^ | 2 | 2 | 0 | 2 | 1 | 2 | 2 | 0 |  |  |  |  | 11/16 |
| Jung, 2011^92^ | 2 | 2 | 1 | 2 | 1 | 2 | 2 | 0 |  |  |  |  | 12/16 |
| Jurina, 2018^93^ | 2 | 2 | 1 | 2 | 1 | 2 | 1 | 0 |  |  |  |  | 11/16 |
| Kanatli, 2017^94^ | 2 | 1 | 1 | 2 | 1 | 2 | 2 | 0 |  |  |  |  | 11/16 |
| Kennedy, 2011^95^ | 1 | 1 | 1 | 1 | 1 | 2 | 2 | 0 |  |  |  |  | 9/16 |
| Kerkhoffs, 2021^15^ | 2 | 2 | 2 | 2 | 1 | 2 | 2 | 0 |  |  |  |  | 13/16 |
| Kim, 2020^96^ | 1 | 2 | 1 | 2 | 1 | 2 | 2 | 0 |  |  |  |  | 11/16 |
| Kim, 2022^201^ | 0 | 2 | 0 | 1 | 1 | 2 | 2 | 0 | 1 | 2 | 1 | 2 | 14/24 |
| Koh, 2022^202^ | 1 | 2 | 0 | 2 | 1 | 1 | 2 | 0 | 2 | 2 | 2 | 2 | 17/24 |
| Kolker, 2004^97^ | 0 | 2 | 1 | 0 | 1 | 2 | 2 | 0 |  |  |  |  | 8/16 |
| Kono, 2006^177^ | 1 | 1 | 1 | 1 | 1 | 2 | 2 | 0 | 2 | 2 | 1 | 0 | 14/24 |
| Korner, 2021^98^ | 2 | 2 | 1 | 1 | 2 | 1 | 0 | 0 |  |  |  |  | 9/16 |
| Koulalis, 2002^99^ | 0 | 1 | 1 | 0 | 1 | 2 | 2 | 0 |  |  |  |  | 7/16 |
| Kreuz, 2006^100^ | 2 | 1 | 1 | 2 | 1 | 2 | 2 | 0 |  |  |  |  | 11/16 |
| Kreuz, 2006^178^ | 1 | 1 | 2 | 1 | 1 | 2 | 2 | 0 | 2 | 2 | 2 | 0 | 16/24 |
| Kubosch, 2016^101^ | 2 | 1 | 0 | 2 | 1 | 2 | 2 | 0 |  |  |  |  | 10/16 |
| Kumai, 2002^102^ | 0 | 1 | 0 | 1 | 0 | 2 | 2 | 0 |  |  |  |  | 6/16 |
| Kwak, 2014^103^ | 1 | 2 | 1 | 2 | 1 | 2 | 1 | 0 |  |  |  |  | 10/16 |
| Lambers, 2020^104^ | 2 | 1 | 1 | 2 | 1 | 2 | 2 | 0 |  |  |  |  | 11/16 |
| Lanham, 2017^179^ | 1 | 1 | 0 | 2 | 1 | 2 | 2 | 0 | 2 | 2 | 2 | 2 | 17/24 |
| Largey, 2009^105^ | 1 | 2 | 0 | 1 | 1 | 2 | 2 | 0 |  |  |  |  | 9/16 |
| Lee, 2008^106^ | 0 | 2 | 0 | 0 | 1 | 2 | 2 | 0 |  |  |  |  | 7/16 |
| Lee, 2013^107^ | 1 | 2 | 2 | 1 | 1 | 2 | 2 | 0 |  |  |  |  | 11/16 |
| Lee, 2015^180^ | 1 | 2 | 2 | 2 | 1 | 2 | 2 | 0 | 2 | 2 | 2 | 2 | 20/24 |
| Lee, 2020^181^ | 2 | 2 | 2 | 2 | 1 | 2 | 2 | 0 | 2 | 2 | 2 | 2 | 21/24 |
| Leumann, 2014^108^ | 2 | 1 | 2 | 2 | 1 | 2 | 2 | 0 |  |  |  |  | 12/16 |
| Li, 2014^109^ | 1 | 1 | 1 | 1 | 1 | 1 | 2 | 0 |  |  |  |  | 8/16 |
| Li, 2021^110^ | 2 | 2 | 1 | 2 | 1 | 2 | 2 | 0 |  |  |  |  | 12/16 |
| Lin, 2010^111^ | 1 | 2 | 0 | 1 | 1 | 2 | 2 | 0 |  |  |  |  | 9/16 |
| Lopez-Alcorocho, 2021^112^ | 1 | 2 | 2 | 1 | 1 | 2 | 2 | 0 |  |  |  |  | 11/16 |
| Lundeen, 2017^113^ | 1 | 1 | 0 | 1 | 1 | 2 | 2 | 0 |  |  |  |  | 8/16 |
| Magnan, 2012^114^ | 0 | 1 | 1 | 0 | 1 | 2 | 2 | 0 |  |  |  |  | 7/16 |
| Manzi, 2021^115^ | 2 | 2 | 1 | 2 | 1 | 2 | 2 | 0 |  |  |  |  | 12/16 |
| Masquijo, 2016^116^ | 2 | 1 | 1 | 2 | 1 | 2 | 2 | 0 |  |  |  |  | 11/16 |
| Mercer, 2022^203^ | 2 | 2 | 0 | 2 | 1 | 2 | 2 | 0 | 2 | 2 | 1 | 2 | 18/24 |
| Ming, 2004^117^ | 1 | 1 | 0 | 1 | 1 | 2 | 2 | 0 |  |  |  |  | 8/16 |
| Minokawa, 2020^118^ | 1 | 2 | 1 | 1 | 1 | 2 | 2 | 0 |  |  |  |  | 10/16 |
| Murphy, 2019^182^ | 1 | 2 | 2 | 1 | 1 | 2 | 2 | 0 | 2 | 0 | 0 | 2 | 15/24 |
| Nakasa, 2019^119^ | 1 | 2 | 1 | 1 | 1 | 2 | 2 | 0 |  |  |  |  | 10/16 |
| Nguyen, 2019^120^ | 1 | 1 | 1 | 1 | 1 | 2 | 2 | 0 |  |  |  |  | 9/16 |
| Ogilvie-Harris, 1999^121^ | 0 | 1 | 1 | 0 | 1 | 2 | 2 | 0 |  |  |  |  | 7/16 |
| Orr, 2017^122^ | 2 | 2 | 1 | 2 | 1 | 2 | 2 | 0 |  |  |  |  | 12/16 |
| Örs, 2022^156^ | 1 | 2 | 0 | 1 | 1 | 2 | 2 | 0 |  |  |  |  | 9/16 |
| Pagliazzi, 2018^124^ | 2 | 1 | 1 | 1 | 1 | 2 | 2 | 0 |  |  |  |  | 10/16 |
| Pagliazzi, 2018^123^ | 2 | 0 | 1 | 2 | 1 | 2 | 2 | 0 |  |  |  |  | 10/16 |
| Park, 2015^183^ | 2 | 2 | 2 | 2 | 1 | 2 | 2 | 0 | 2 | 2 | 0 | 2 | 19/24 |
| Park, 2018^184^ | 2 | 2 | 1 | 2 | 1 | 2 | 2 | 0 | 2 | 2 | 2 | 2 | 20/24 |
| Park, 2021^125^ | 2 | 1 | 1 | 2 | 1 | 2 | 2 | 0 |  |  |  |  | 11/16 |
| Paul, 2009^126^ | 2 | 2 | 0 | 2 | 1 | 1 | 1 | 0 |  |  |  |  | 9/16 |
| Penner, 2021^127^ | 0 | 0 | 1 | 0 | 1 | 1 | 2 | 0 |  |  |  |  | 5/16 |
| Petersen, 2014^128^ | 2 | 2 | 2 | 2 | 1 | 2 | 2 | 0 |  |  |  |  | 13/16 |
| Rak Choi, 2021^185^ | 1 | 2 | 1 | 1 | 1 | 2 | 2 | 0 | 2 | 2 | 1 | 2 | 17/24 |
| Reilingh, 2016^187^ | 1 | 2 | 2 | 2 | 1 | 2 | 2 | 2 | 2 | 2 | 2 | 2 | 22/24 |
| Reilingh, 2018^186^ | 1 | 1 | 2 | 2 | 1 | 2 | 2 | 0 | 2 | 1 | 1 | 2 | 17/24 |
| Richter, 2022^157^ | 1 | 2 | 1 | 1 | 0 | 0 | 2 | 0 |  |  |  |  | 7/16 |
| Ross, 2016^188^ | 1 | 2 | 1 | 2 | 1 | 2 | 2 | 0 | 2 | 2 | 1 | 1 | 17/24 |
| Sabaghzadeh, 2020^129^ | 1 | 2 | 1 | 1 | 1 | 2 | 2 | 0 |  |  |  |  | 10/16 |
| Sadlik, 2015^130^ | 0 | 2 | 0 | 1 | 1 | 2 | 2 | 0 |  |  |  |  | 8/16 |
| Sadlik, 2019^189^ | 2 | 2 | 2 | 2 | 1 | 2 | 2 | 0 | 2 | 2 | 1 | 2 | 20/24 |
| Sammarco, 2002^132^ | 2 | 2 | 1 | 2 | 1 | 2 | 2 | 0 |  |  |  |  | 12/16 |
| Schafer, 2022^158^ | 1 | 2 | 0 | 1 | 0 | 2 | 2 | 0 |  |  |  |  | 8/16 |
| Schneider, 2009^133^ | 1 | 2 | 1 | 2 | 1 | 2 | 2 | 0 |  |  |  |  | 11/16 |
| Schuh, 2004^134^ | 1 | 2 | 0 | 1 | 1 | 2 | 2 | 0 |  |  |  |  | 9/16 |
| Schuman, 2002^135^ | 1 | 2 | 2 | 2 | 1 | 2 | 2 | 0 |  |  |  |  | 12/16 |
| Scranton, 2006^136^ | 1 | 0 | 0 | 2 | 1 | 2 | 2 | 0 |  |  |  |  | 8/16 |
| Shang, 2016^190^ | 2 | 2 | 2 | 2 | 1 | 2 | 2 | 0 | 2 | 2 | 2 | 2 | 21/24 |
| Shimozono, 2018^191^ | 2 | 2 | 1 | 2 | 1 | 2 | 2 | 0 | 2 | 2 | 2 | 2 | 20/24 |
| Shimozono, 2019^193^ | 2 | 2 | 1 | 2 | 1 | 2 | 2 | 0 | 2 | 2 | 1 | 2 | 19/24 |
| Shimozono, 2021^192^ | 2 | 2 | 1 | 2 | 1 | 2 | 2 | 0 | 2 | 2 | 1 | 2 | 19/24 |
| Struckmann, 2020^194^ | 1 | 2 | 2 | 2 | 1 | 2 | 2 | 0 | 2 | 2 | 2 | 2 | 20/24 |
| Tan, 2022^204^ | 2 | 2 | 0 | 1 | 1 | 1 | 2 | 0 | 2 | 2 | 0 | 0 | 13/24 |
| Tahta, 2017^195^ | 2 | 2 | 0 | 2 | 1 | 2 | 2 | 0 | 1 | 2 | 2 | 2 | 18/24 |
| Takao, 2004^196^ | 2 | 2 | 2 | 2 | 1 | 2 | 2 | 0 | 2 | 2 | 1 | 0 | 18/24 |
| Taranow, 1999^137^ | 1 | 2 | 1 | 1 | 1 | 2 | 2 | 0 |  |  |  |  | 10/16 |
| Tosun, 2021^138^ | 2 | 2 | 0 | 1 | 1 | 2 | 2 | 0 |  |  |  |  | 10/16 |
| Usuelli, 2018^139^ | 2 | 2 | 2 | 2 | 1 | 2 | 2 | 0 |  |  |  |  | 13/16 |
| Valderrabano, 2013^140^ | 2 | 2 | 1 | 2 | 1 | 2 | 2 | 0 |  |  |  |  | 12/16 |
| Van Bergen, 2013^141^ | 2 | 2 | 1 | 2 | 1 | 2 | 2 | 0 |  |  |  |  | 12/16 |
| Vannini, 2017^142^ | 2 | 2 | 1 | 2 | 1 | 2 | 2 | 0 |  |  |  |  | 12/16 |
| Ventura, 2013^143^ | 2 | 2 | 1 | 2 | 1 | 2 | 2 | 0 |  |  |  |  | 12/16 |
| Volpi, 2014^144^ | 1 | 0 | 1 | 1 | 1 | 0 | 2 | 0 |  |  |  |  | 6/16 |
| Vuurberg, 2018^28^ | 2 | 2 | 2 | 2 | 1 | 2 | 2 | 0 |  |  |  |  | 13/16 |
| Wan, 2022^159^ | 1 | 2 | 0 | 1 | 0 | 2 | 2 | 0 |  |  |  |  | 8/16 |
| Wang, 2022^160^ | 1 | 2 | 0 | 1 | 1 | 2 | 2 | 0 |  |  |  |  | 9/16 |
| Walther, 2014^145^ | 1 | 1 | 1 | 1 | 1 | 2 | 2 | 0 |  |  |  |  | 9/16 |
| Wang, 2019^146^ | 2 | 1 | 1 | 2 | 1 | 2 | 2 | 0 |  |  |  |  | 11/16 |
| Wei, 2022^205^ | 1 | 2 | 2 | 1 | 2 | 2 | 2 | 0 | 2 | 2 | 2 | 2 | 20/24 |
| Weigelt, 2019^29^ | 2 | 2 | 1 | 2 | 1 | 2 | 1 | 0 |  |  |  |  | 11/16 |
| Whittaker, 2005^147^ | 0 | 0 | 1 | 0 | 1 | 2 | 2 | 0 |  |  |  |  | 6/16 |
| Windhofer, 2022^161^ | 2 | 2 | 0 | 2 | 0 | 2 | 2 | 0 |  |  |  |  | 10/16 |
| Woefle, 2013^148^ | 0 | 2 | 0 | 0 | 1 | 2 | 2 | 0 |  |  |  |  | 7/16 |
| Yang, 2020^149^ | 2 | 2 | 1 | 2 | 1 | 2 | 2 | 0 |  |  |  |  | 12/16 |
| Yontar, 2022^162^ | 2 | 2 | 0 | 2 | 0 | 2 | 2 | 0 |  |  |  |  | 10/16 |
| Yoon, 2014^197^ | 2 | 2 | 1 | 2 | 1 | 2 | 2 | 0 | 1 | 2 | 2 | 2 | 19/24 |
| Yoshimura, 2013^198^ | 2 | 2 | 0 | 2 | 1 | 2 | 2 | 0 | 2 | 2 | 2 | 2 | 19/24 |
| Younger, 2016^150^ | 2 | 1 | 2 | 2 | 1 | 1 | 2 | 0 |  |  |  |  | 11/16 |
| Zhang, 2021^151^ | 2 | 2 | 1 | 2 | 1 | 2 | 2 | 0 |  |  |  |  | 12/16 |
| Zhao, 2022^206^ | 1 | 2 | 0 | 1 | 1 | 0 | 2 | 0 | 2 | 2 | 2 | 2 | 15/24 |
| Zhu, 2019^152^ | 1 | 0 | 1 | 1 | 1 | 2 | 2 | 0 |  |  |  |  | 8/16 |

# Supplementary files

Supplementary file 1: Forest plot complication rates

Supplementary file 2: Forest plot Grade 1 complications

Supplementary file 3: Forest plot Grade 2 complications

Supplementary file 4: Forest plot Grade 3 complications
