# Supplementary figures and images for: The Frequency and Severity of Complications in Surgical Treatment of Osteochondral Lesions of the Talus: A Systematic Review and Meta-Analysis of 6,962 Lesions
Source: Cartilage. 2023 Mar 9;14(2):180–97. doi: 10.1177/19476035231154746 (PMC10416205; doi:10.1177/19476035231154746)

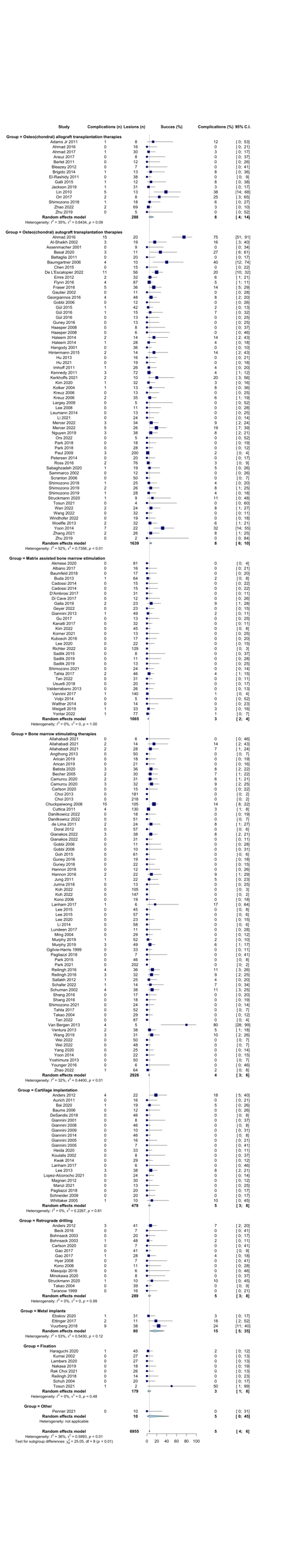

Supplement: sj-jpg-2-car-10.1177_19476035231154746 – Supplemental material for The Frequency and Severity of Complications in Surgical Treatment of Osteochondral Lesions of the Talus: A Systematic Review and Meta-Analysis of 6,962 Lesions [file sj-jpg-2-car-10.1177_19476035231154746.jpg]

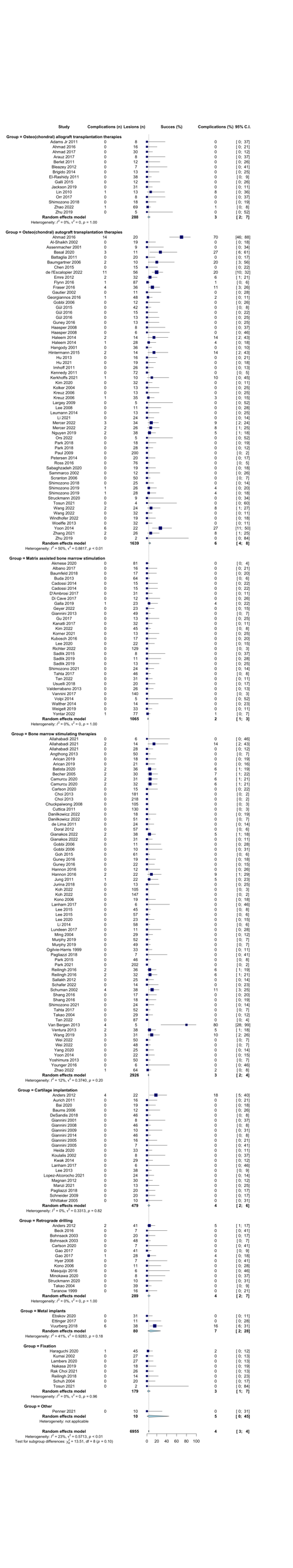

Supplement: sj-jpg-3-car-10.1177_19476035231154746 – Supplemental material for The Frequency and Severity of Complications in Surgical Treatment of Osteochondral Lesions of the Talus: A Systematic Review and Meta-Analysis of 6,962 Lesions [file sj-jpg-3-car-10.1177_19476035231154746.jpg]

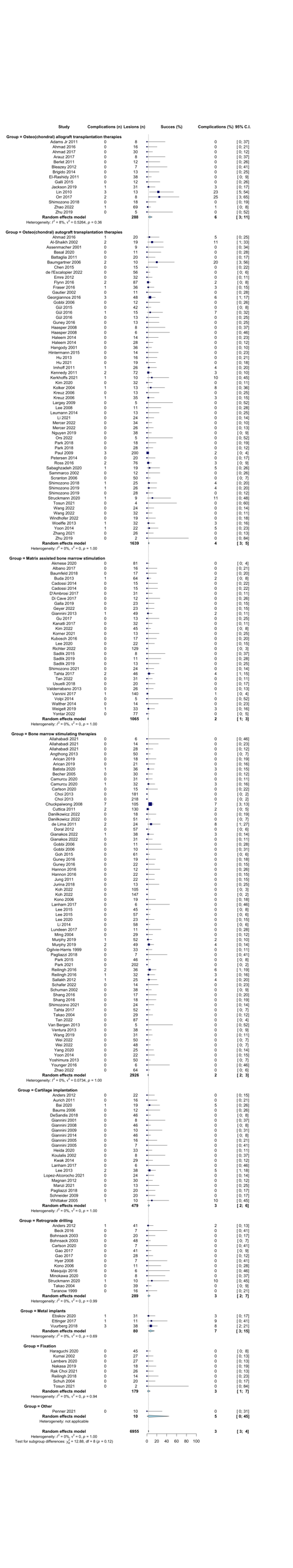

Supplement: sj-jpg-4-car-10.1177_19476035231154746 – Supplemental material for The Frequency and Severity of Complications in Surgical Treatment of Osteochondral Lesions of the Talus: A Systematic Review and Meta-Analysis of 6,962 Lesions [file sj-jpg-4-car-10.1177_19476035231154746.jpg]

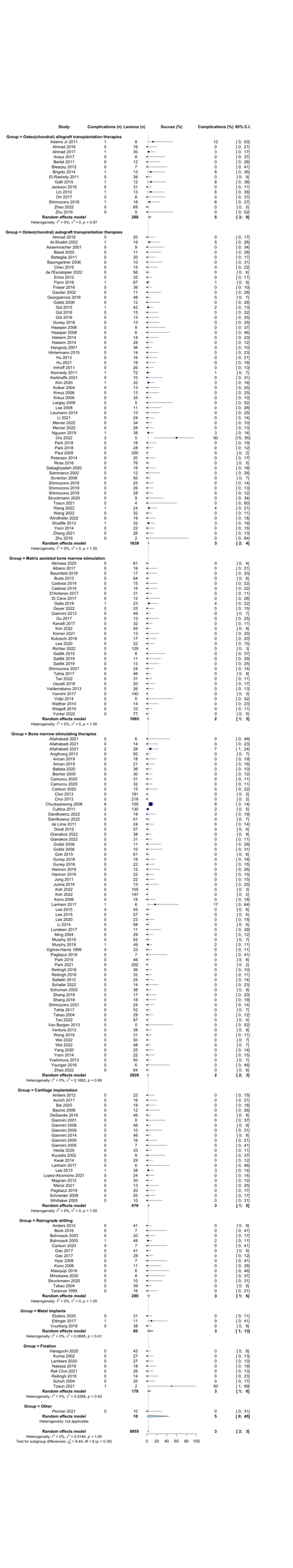

Supplement: sj-jpg-5-car-10.1177_19476035231154746 – Supplemental material for The Frequency and Severity of Complications in Surgical Treatment of Osteochondral Lesions of the Talus: A Systematic Review and Meta-Analysis of 6,962 Lesions [file sj-jpg-5-car-10.1177_19476035231154746.jpg]
